# High concordance of human immunodeficiency virus-1 genotypic drug resistance generated from paired cerebrospinal fluid and plasma in antiretroviral therapy -naive or -experienced patients

**DOI:** 10.3389/fmicb.2025.1518225

**Published:** 2025-03-26

**Authors:** Xizi Deng, Jiaojiao Li, Ruiying He, Yingfen Wen, Yaqing Lin, Liya Li, Xuemei Ling, Fengyu Hu, Linghua Li, Yun Lan

**Affiliations:** ^1^Institute of Infectious Diseases, Guangzhou Eighth People’s Hospital, Guangzhou Medical University, Guangzhou, China; ^2^Infectious Disease Center, Guangzhou Eighth People’s Hospital, Guangzhou Institute of Clinical Infectious Diseases, Guangzhou Medical University, Guangzhou, China

**Keywords:** HIV-1, CSF, plasma, drug resistance, genotype

## Abstract

**Background:**

The development of human immunodeficiency virus (HIV) drug resistance significantly impairs patients’ quality of life. However, the HIV-1 drug resistance patterns in the central nervous system (CNS) have been poorly studied.

**Objective:**

We aimed to compare HIV-1 genotypes and drug resistance mutations (DRMs) derived from the cerebrospinal fluid (CSF) and plasma of antiretroviral therapy (ART)-naive or -experienced patients.

**Methods:**

The matched CSF and plasma samples from 59 patients with HIV were subjected to HIV proteinase (PR), reverse transcriptase (RT), and integrase (IN) gene sequencing. To determine the HIV-1 genotypes, sequences were assessed with the Context-based Modelling for Expeditious Typing (COMET) tool, and the neighbour-joining (NJ) phylogenetic tree was used to confirm the results. Quality control based on genotype and phylogenetic tree analysis was conducted to assess potential sequence contamination during the detection process. The HIV-1 drug resistance database of Stanford University was used to identify DRMs and sensitivity to four drug classes [protease inhibitors (PIs), nucleoside reverse transcriptase inhibitors (NRTIs), nonnucleoside reverse transcriptase inhibitors (NNRTIs), and integrase strand transfer inhibitors (INSTIs)].

**Results:**

Of the 59 patients with HIV with matched CSF and plasma samples, samples from 37 were included in the study after excluding the samples that failed to be successfully amplified. CRF01_AE was the most frequently occurring genotype, with a frequency of 46.0% (17/37), followed by CRF07_BC (27.0%, 10/37) and CRF55_01B (10.8%, 4/37). Among the 37 patients, 37.8% (14/37) carried at least one DRM, and the mutation sites were consistent in both CSF and matched plasma, except one. NNRTI-related resistance mutations were the predominant DRMs, particularly V179D/E, present in 71.4% (10/14) of patients with DRM sites, primarily in ART-naive patients.

**Conclusion:**

A high concordance of HIV-1 DRMs between CSF and plasma samples was observed. No unique mutations were identified in CSF other than those in plasma, indicating that the mutant variants in CSF were derived from blood.

## Introduction

The central nervous system (CNS) is an important site of human immunodeficiency virus (HIV) replication and persistence (Honeycutt et al., 2018). HIV-1 may invade the CNS through infected CD4^+^ T cells that cross the blood–brain barrier (BBB) soon after systemic infection (Sturdevant et al., 2015). HIV-1 entering the CNS can establish a viral neural reservoir by infecting and adapting to microglia (a brain-specific macrophage population) with low expression of CD4, leading to the evolution of compartmentalized HIV-1 variants in the CNS (Joseph et al., 2019; Chen et al., 2022; Ko et al., 2019). The ability of antiretroviral drugs to penetrate the BBB varies. Findings from several studies have suggested that concentrations of particular drugs may be insufficient to suppress viral replication in the CNS (Asahchop et al., 2017; Moreno-Gamez et al., 2015). The limited penetration of some antiretroviral drugs into the CNS through the BBB, combined with incomplete adherence to ART and ART interruption, may contribute to the compartmentalization of HIV-1 drug resistance between the CSF and plasma, which ultimately results in therapeutic failure (Gianella et al., 2016; Kincer et al., 2023; Vissers et al., 2010; Cysique and Brew, 2009). Findings from previous studies have indicated that drug resistance can develop differently in the two compartments and that the CSF can serve as a viral reservoir for drug resistance mutations (DRMs). In addition, significant differences in the relative frequencies of certain DRMs, such as M184I/V, between CSF and plasma have been reported (Bergroth et al., 2009; Stingele et al., 2001; Kelentse et al., 2020). DRMs in the CSF could evade routine blood diagnostics. Consequently, therapeutic success may be compromised if treatment is adjusted solely based on genotypic resistance patterns detected in one compartment. Currently, HIV-1 drug resistance patterns in the CNS have been poorly studied in China. Therefore, in this study, we aimed to compare HIV-1 genotypes and DRMs derived from the CSF and plasma of ART-naive and ART-experienced patients.

## Materials and methods

### Patients and data collection

We retrospectively collected matched CSF and plasma samples from 59 ART-naive or ART-experienced patients with HIV who were hospitalized at Guangzhou Eighth People’s Hospital from February 2018 to February 2019. ART-naive patients were patients who have had no prior exposure to any antiretroviral drugs or treatment regimens; ART-experienced patients were patients previously treated with ART and currently taking ART who have experienced virological failure. CSF and plasma samples were obtained for HIV protease (PR), reverse transcriptase (RT), and integrase (IN) gene sequencing. Epidemiological data for the patients (including age, sex, transmission route, year at diagnosis, and treatment regimen) were downloaded from the National Free Antiretroviral Treatment Database for Disease Control and Prevention in China. The Clinical data (HIV-1 RNA viral load, total protein amount in CSF, CSF glucose level, CSF white blood cell count, CSF chloride level, and plasma CD4^+^/CD8^+^ T-cell counts) of these patients were collected and anonymously analyzed.

The study was approved by the Institutional Review Board of Guangzhou Eighth People’s Hospital, and all patients signed informed consent forms.

### RNA extraction, amplification, sequencing and sequence splicing

Viral RNA was extracted from 200 μL CSF and plasma samples with an automatic magnetic bead-based Virus RNA Extraction Kit (Daan, China) according to the manufacturer’s instructions. Amplification of the entire PR gene plus partial RT gene (PRRT region, HXB2 2,253–3,318, covering all 99 amino acids of PR and the first 240 amino acids of RT) and entire IN gene (IN region, HXB2 4,230–5,093, covering all 288 amino acids of IN) was performed with an in-house RT polymerase chain reaction (RT–PCR) procedure, as previously described (Lan et al., 2022; Lan et al., 2020). The amplified PCR products were separated by 1% agarose gel electrophoresis and the positive products were sent for Sanger sequencing (Tianyi Huiyuan Genomics Company, China). The obtained sequences were spliced with Sequencher analysis software (Version 5.4.6) and then aligned using BioEdit software (Version 7.2).

### HIV-1 genotyping and phylogenetic analysis

The sequences were aligned with 2013 HIV-1 subtyping reference strains downloaded from the Los Alamos HIV Sequence Database via BioEdit software. To determine the HIV-1 genotypes, sequences were assessed with the Context-based Modelling for Expeditious Typing (COMET) tool (Struck et al., 2014). The neighbour-joining (NJ) phylogenetic tree was used for confirmation. The phylogenetic tree was constructed via the NJ method with the Kimura two-parameter model with the MEGA program 6.0(Tamura et al., 2013), and the reliability of the tree topologies was assessed by bootstrapping with 1,000 replications. The pairwise genetic distance between CSF and plasma sequences was calculated using the MEGA program.

Quality control of the sequences derived from CSF and blood was conducted through genotyping and phylogenetic analysis. Genotype consistency between CSF and plasma was evaluated. Sequences were considered reliable for DRMs and drug resistance (DR) if the genotypes matched and CSF and plasma sequences clustered in the phylogenetic tree, thus excluding contamination. Inconsistent sequences were excluded from further analysis.

### DRMs and DR analysis

The HIV-1 drug resistance database of Stanford University (HIVdb version 9.1, https://hivdb.stanford.edu/hivdb/by-sequences/) was used to identify DRMs and sensitivity to four drug classes [protease inhibitors (PIs), nucleoside reverse transcriptase inhibitors (NRTIs), nonnucleoside reverse transcriptase inhibitors (NNRTIs) or integrase strand transfer inhibitors (INSTIs)]. Sequences associated with low-level, intermediate, or high-level resistance categories were defined as conferring drug resistance.

### Statistical analysis

All the statistical analyses were performed using the IBM SPSS program, version 25.0. Qualitative variables are presented as percentages (%), and quantitative variables are presented as medians and interquartile ranges (IQRs) or ranges.

## Results

### Gene sequencing and demographic characteristics

Among all the 59 patients with HIV with matched CSF and plasma samples, 4 CSF samples had low viral loads and could not be tested for drug resistance. Among the remaining 55 CSF samples, PRRT sequences were obtained for 49 (89.1%), and IN sequences were obtained for 48 (87.3%). Among all 59 plasma samples, PRRT sequences were obtained for 56 (94.9%), and IN sequences were obtained for 54 (91.5%). Finally, 37 patients with HIV with matched CSF and plasma PRRT and IN sequences were included in this study, including 27 ART-naive patients and 10 ART-experienced patients. The age of the patients ranged from 25 to 73 years, with a median (IQR) age of 45(30–53) years. In total, 81.1% (30/37) of the patients were male. Approximately two-thirds of the patients had confirmed infections in 2018. Heterosexual (HET) contact was the predominant risk group (51.4%, 19/37), followed by men who have sex with men (MSM) (21.6%, 8/37) and people who inject drugs (5.4%, 2/37). The median CSF and plasma HIV-1 RNA viral load at the time of the drug resistance test were 4.89 and 4.95 (log 10, copies/mL), respectively. All of the ART-experienced patients were treated with two NRTIs, with 60.0% of whom were treated with NNRTIs and 20.0% of whom were treated with PIs or INSTIs. The demographic characteristics of the patients are summarized in Table 1.

**Table 1:** The demographic characteristics of the patients.

Characteristic	Total	ART-naive patients	ART-experienced patients
Number	37	27	10
Age, years, median (IQR)	45 (30–53)	47 (34–54)	31 (27–45)
Male, number (%)	30 (81.08%)	22 (81.48%)	8 (80.00%)
White blood cell count of CSF,10^6^/L, median (IQR)	11 (4–48)	11 (4–71)	9 (3–15)
Proteins of CSF, mg/L, median (IQR)	926.6 (567.2–1711.5)	708.7 (527.4–1746)	1076.5 (833.7–1439.3)
Cerebrospinal fluid glucose, mmol/L, median (IQR)	2.81 (2.06–3.58)	2.9 (2.26–3.54)	2.5 (2.02–3.47)
Cerebrospinal fluid chloride,mmol/L, median (IQR)	115.3 (112.9–119.3)	115.3 (112.6–119.7)	114.3 (113.15–118.38)
CSF HIV-1 RNA, log10 copies/mL, median (IQR)	4.89 (4.46–5.42)	4.89 (4.48–5.47)	4.84 (4.15–5.23)
Plasma HIV-1 RNA, log10 copies/mL, median (IQR)	4.95 (4.49–5.62)	5.12 (4.77–5.44)	4.76 (3.55–5.71)
Baseline CD4 count, cells/mm^3^, median (IQR)	105 (16–245)	105 (16–245)	99 (15–217)
Baseline CD8 count, cells/mm^3^, median (IQR)	714 (366–881)	714 (366–988)	652 (377–790)
CD4 count at sampling time, cells/mm^3^, median (IQR)	48 (14–153)	48 (14–160)	48 (28–92)
CD8 count at sampling time, cells/mm3, median (IQR)	480 (316–799)	565 (313–915)	361 (348–743)
**Transmission route, *n* (%)**			
Heterosexual	19 (51.35)	-	19 (51.35)
Men who have sex with men	8 (21.62)	-	8 (21.62)
People who inject drugs	2 (5.41)	-	2 (5.41)
Unknown	8 (21.62)	-	8 (21.62)
**Initial treatment, *n* (%)**			
PI+2NRTIs	2 (5.41)	-	2 (20.00)
NNRTI+2NRTIs	6 (16.22)	-	6 (60.00)
INSTI+2NRTIs	2 (5.41)	-	2 (20.00)
Time under ART, months, median (IQR)	14.47 (9.43–28.77)	-	14.47 (9.43–28.77)

### HIV-1 genotypes and phylogenetic analysis

**Figure 1:**
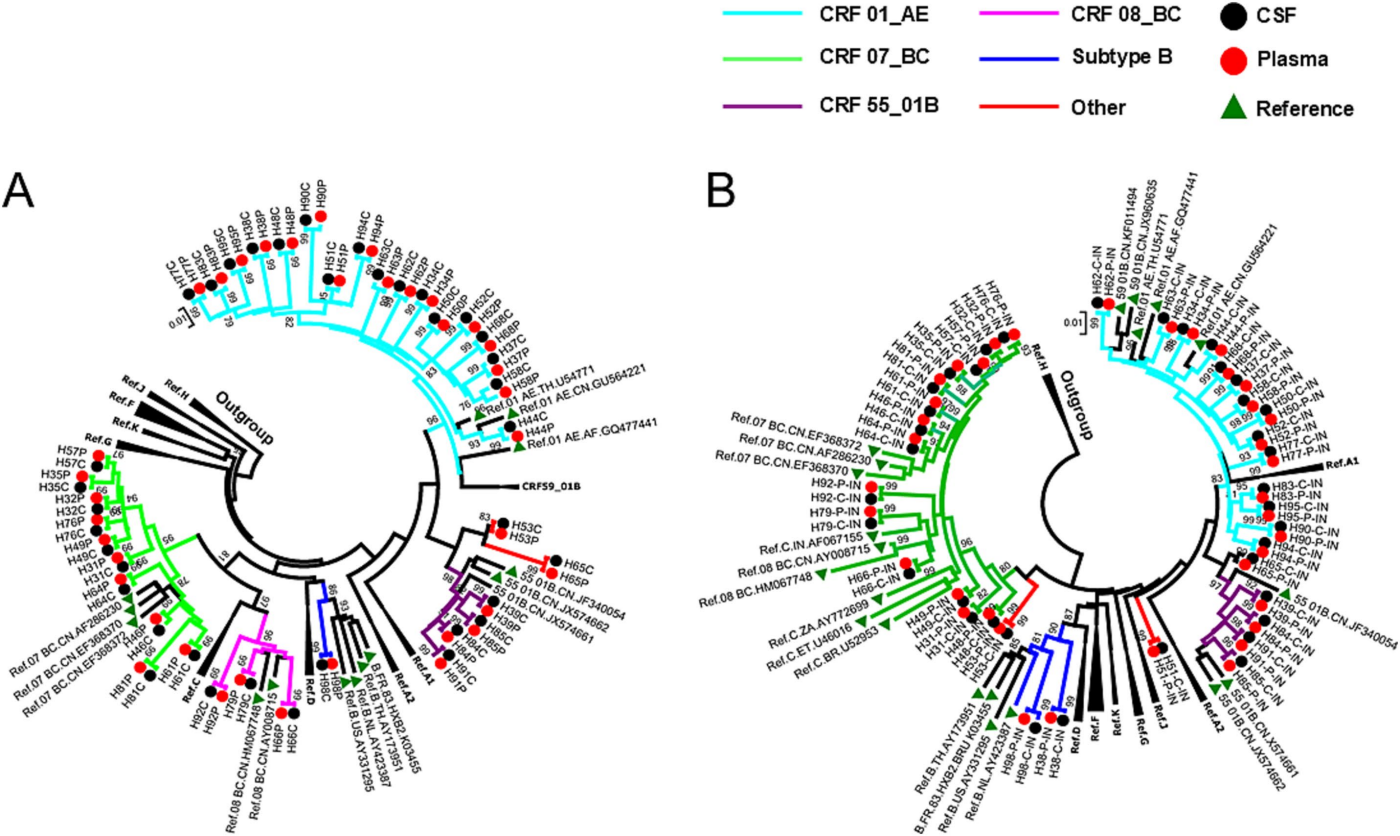
Neighbour-joining (NJ) phylogenetic tree based on protease (PR) gene plus partial reverse transcriptase (RT) gene (PRRT) sequences **(A)** or integrase (IN) sequences **(B)** obtained from 37 patients whose main genotypes were initially identified by Context-based modelling for expeditious typing (COMET) of human immunodeficiency virus (HIV). Reference sequences were downloaded from the Los Alamos HIV Sequence Database (https://www.hiv.lanl.gov/content/index); subtype H sequences were selected as the outgroup. Genetic distances were calculated for all patients whose drug-resistance mutation (DRM) sites were removed from paired cerebrospinal fluid (CSF)–plasma PRRT or IN genes. The median (range) genetic distance of the PRRT sequences was 0.0% (0.0–0.9%), which was less than 0.1% for all patients except for two (0.1 and 0.9%). The median (range) genetic distance of the IN sequences was 0.0% (0.0–0.9%) and was less than or equal to 0.1% for all patients except for five (all 0.1%).

According to COMET HIV-1 analysis based on PRRT sequences, HIV genotypes identified in the 37 matched CSF and plasma samples were identical: CRF01_AE was the most frequently occurring genotype, with a frequency of 46.0% (17/37), followed by CRF07_BC (27.0%, 10/37) and CRF55_01B (10.8%, 4/37). A total of five genotypes or circulating recombinant forms (CRFs) were confirmed (94.6%, 35/37) according to the NJ phylogenetic tree based on 37 matched CSF and plasma PRRT sequences (Figure 1A), which was constructed to determine the evolutionary relationship of these sequences. In addition, 2 recombinant strains were observed (COMET tool ‘unassigned’ and not clustered with any reference sequences by the phylogenetic tree), which were classified as ‘other’ genotypes (Figure 1A). CRF07_BC, CRF08_BC, and genotype C cannot fall into clusters on the basis of IN sequences, as they lack the necessary breakpoints for genotyping (Figure 1B). Genetic distances were calculated for all patients with paired CSF-plasma PRRT or IN genes after removing the DRMs. The median genetic distance for the PRRT sequences was 0.0%, ranging from 0.0 to 0.9%, except for two patients whose distances were 0.1 and 0.9%, respectively. For IN sequences, the median genetic distance was also 0.0%, with a range of 0.0 to 0.9%, and all but five patients had distances less than or equal to 0.1%, with those five patients all having a distance of 0.1%.

### DRMs and DR differences between CSF and plasma

Among the 37 patients, 14 (37.8%) carried at least one DRM. Among them, 71.4% (10/14) were ART-naive patients, whereas 28.6% (4/14) were ART-experienced patients (Table 2).

**Table 2:** Characteristics of patients with drug resistance mutations sites.

No.	Sex	Age	Months since HIV diagnosis	Months since ART	ART regimen	Subtypes based on PRRT		HIV-1 VL (Log10, copies/mL)		PI resistance mutations		NRTI resistance mutations		NNRTI resistance mutations		INSTI resistance mutations	
						CSF	Plasma	CSF	Plasma	CSF	Plasma	CSF	Plasma	CSF	Plasma	CSF	Plasma
H31	M	56	0	0		07_BC	07_BC	7.00	5.99	-	-	-	-	V179D	V179D	-	-
H39	M	48	0	0		55_01B	55_01B	6.06	4.77	-	-	-	-	V179E	V179E	-	-
H46	M	71	0	0		07_BC	07_BC	5.37	4.47	-	-	-	-	V179E	V179E	-	-
H52	F	28	11.33	11	EFV + 3TC + TDF	01_AE	01_AE	5.60	4.07	-	-	K65R	K65R	L100I,K103N,G190S	L100I,K103N,G190S	-	-
H53	M	30	0	0		Other	Other	5.00	5.26	-	-	-	-	V179E	V179E	-	-
H62	M	53	0.20	0		01_AE	01_AE	4.42	4.87	-	-	-	-	A98G	A98G	-	G163R
H63	M	47	0.10	0		01_AE	01_AE	4.46	4.12	-	-	S68G	S68G	-	-	L74M	L74M
H65	M	28	73.83	73	EFV + 3TC + D4T	Other	Other	4.12	5.29	-	-	-	-	V179E	V179E	-	-
H77	M	33	30.43	30	EFV + 3TC + TDF	01_AE	01_AE	4.88	4.69	-	-	-	-	-	-	A128T	A128T
H81	F	51	0	0		07_BC	07_BC	4.56	4.48	-	-	T215S	T215S	V179D	V179D	-	-
H84	M	34	0.23	0		55_01B	55_01B	4.90	4.94	-	-	-	-	V179E	V179E	-	-
H85	M	38	0.27	0		55_01B	55_01B	3.60	5.72	-	-	-	-	E138G,V179E	E138G,V179E	-	-
H90	M	26	25.33	25	EFV + 3TC + TDF	01_AE	01_AE	4.12	3.38	-	-	D67N,K70E,M184V	D67N,K70E,M184V	V106I,V179D	V106I,V179D	-	-
H91	M	21	1.57	0		55_01B	55_01B	5.49	5.18	-	-	-	-	V179E	V179E	-	-

M, male; F, female; EFV, efavirenz; TDF, tenofovir disoproxil fumarate; 3TC, lamivudine; PI, protease inhibitors; NRTI, nucleotide reverse transcriptase inhibitors; NNRTI, non-nucleotide reverse transcriptase inhibitors; INSTI, integrase strand-transfer inhibitors.

Among the 14 patients with DRM sites, the CSF and plasma genotypes were identical [CRF01_AE (35.7%, 5/14), CRF55_01B (28.6%, 4/14) and CRF07_BC (21.4%, 3/14)], the mutation sites were consistent except in one patient (the IN drug mutation G163R was present in the plasma only for one ART-naive patient), and approximately half of the patients (42.9%, 6/14) had more than one mutation site. The DRMs identified were mainly NNRTI, NRTI, and IN resistance mutations; no PI resistance mutations were found. The NRTI-related resistance mutation sites were K65R, D67N, S68G, K70E, M184V and T215S (all 7.1%, 1/14); the NNRTI-related resistance mutation sites were mainly V179D/E (71.4%, 10/14), A98G, L100I, K103N, V106I, E138G, and G190S (all 7.1%, 1/14); and the IN-related resistance mutation sites were L74M, A128T and G163R (all 7.1%, 1/14) (Table 2).

Five of the 14 patients with DRM sites exhibited varying levels of antiviral drug resistance, with four demonstrating resistances to antiviral agents currently used in China. Notably, patient H81 harbored a resistance mutation that confers resistance to D4T, a drug not currently in use within the country (Table 3).

**Table 3:** Drug resistance profiles in CSF and plasma of patients resistant to antiviral drugs.

Patient ID	Specimen type	PI resistance								NRTI resistance							NNRTI resistance					IN resistance				
		ATV/r	DRV/r	FPV/r	IDV/r	LPV/r	NFV	SQV/r	TPV/r	ABC	AZT	D4T	DDI	FTC	3TC	TDF	DOR	EFV	ETR	NVP	RPV	BIC	CAB	DTG	EVG	RAL
H52	CSF	S	S	S	S	S	S	S	S	M	S	H	H	M	M	M	M	H	M	H	H	S	S	S	S	S
H52	Plasma	S	S	S	S	S	S	S	S	M	S	H	H	M	M	M	M	H	M	H	H	S	S	S	S	S
H62	CSF	S	S	S	S	S	S	S	S	S	S	S	S	S	S	S	L	L	P	M	L	S	S	S	S	S
H62	Plasma	S	S	S	S	S	S	S	S	S	S	S	S	S	S	S	L	L	P	M	L	S	S	S	L	L
H81	CSF	S	S	S	S	S	S	S	S	S	P	L	P	S	S	S	S	P	P	P	P	S	S	S	S	S
H81	Plasma	S	S	S	S	S	S	S	S	S	P	L	P	S	S	S	S	P	P	P	P	S	S	S	S	S
H85	CSF	S	S	S	S	S	S	S	S	S	S	S	S	S	S	S	S	L	L	L	L	S	S	S	S	S
H85	Plasma	S	S	S	S	S	S	S	S	S	S	S	S	S	S	S	S	L	L	L	L	S	S	S	S	S
H90	CSF	S	S	S	S	S	S	S	S	M	S	M	M	H	H	L	P	M	L	M	M	S	S	S	S	S
H90	Plasma	S	S	S	S	S	S	S	S	M	S	M	M	H	H	L	P	M	L	M	M	S	S	S	S	S

The drugs highlighted in red are currently used in China.

S, susceptible; P, potential low-level resistance; L, low-level resistance; M, intermediate resistance; H, high-level resistance; PI, protease inhibitors; NRTI, nucleotide reverse transcriptase inhibitors; NNRTI, non-nucleotide reverse transcriptase inhibitors; INSTI, integrase strand-transfer inhibitors; ATV/r, atazanavir/ritonavir; DRV/r, darunavir/ritonavir; FPV/r, fosamprenavir/ritonavir; IDV/r, indinavir/ritonavir;LPV/r, Lopinavir/ritonavir; NFV, nelfinavir; SQV/r, saquinavir/ritonavir; TPV/r, tipranavir/ritonavir; ABC, abacavir; AZT, azidothymidine; D4T, stavudine; DDI, didanosine; FTC, emtricitabine; 3TC, lamivudine; TDF, tenofovir disoproxil fumarate; DOR, doravirine; EFV, efavirenz; ETR, etravirine; NVP, nevirapine; RPV, rilpivirine; BIC, bictegravir; CAB, cabotegravir; DTG, dolutegravir; EVG, elvitegravir; RAL, raltegravir.

## Discussion

In this study, we conducted a comparative analysis of PI, NRTI, NNRTI, and INSTI resistance profiles in the CSF and plasma of patients with HIV, irrespective of their ART status.

The COMET HIV-1 and the phylogenetic tree analysis of PRRT sequences revealed consistent findings regarding HIV-1 genotypes in CSF and plasma, thereby validating the reliability of the genotyping results. Specifically, we found that CRF01_AE, CRF07_BC, and CRF55_01B were the dominant genotypes, and these genotypes are consistent with the predominant genotypes currently circulating in Guangdong Province.(Zhou et al., 2019).

Our study reveals a high concordance of HIV-1 DRMs between CSF and plasma, with no mutations identified in CSF that were absent in plasma. The similar phenomena were also observed in a study with 62 patients conducted in Chongqing, China, where the major stain was CRF07_BC (64.5%) (Wang et al., 2023). The two studies conducted in Chongqing and Guangzhou consistently indicated that few patients carried additional DRMs in plasma, and no mutations were found in the CSF that were not already present in the plasma. We identified NNRTI-related resistance mutations as the predominant DRMs, particularly V179D/E, which were present in 71.4% (10/14) of patients with DRM sites, primarily in ART-naive patients. This observation underscores the importance of monitoring DRMs in HIV CRFs before initiating ART and highlights the urgent need for effective preventive measures to reduce transmission. Furthermore, we identified one patient who exhibited an additional HIV-1 DRM, G163R, in the plasma. G163R/K are nonpolymorphic in all subtypes except subtype F. These mutations have been predominantly identified in individuals who are undergoing treatment with raltegravir (RAL). These mutations seem to be accessory mutations since they commonly cooccur with other INSTI resistance mutations, particularly N155H. However, the associated phenotypic effects have not been thoroughly characterized (Hachiya et al., 2017; Margot et al., 2019; Sax et al., 2011). Retrospective analysis of the clinical data from patients harbouring the G163R mutation revealed that the patient had no prior exposure to any antiretroviral drugs or treatment regimens, suggesting the possibility of transmitted drug resistance. Additionally, it was hypothesized that the frequency of quasispecies harbouring the G163R mutation was relatively low and that the insufficient sequencing depth of Sanger sequencing may have contributed to the failure to detect this mutation in CSF samples.

Our research has certain limitations, primarily stemming from the limited sample size. The study population consisted of patients with detectable HIV viral loads in plasma and clinical indications for lumbar puncture during the study period, representing a highly specific subset of individuals living with HIV in China. Consequently, the generalizability of our findings to the broader population of individuals living with HIV in China may be limited. Moreover, the inadequate depth of Sanger sequencing may have resulted in the oversight of certain low-abundance DRMs. Therefore, further studies with larger sample sizes and next-generation sequencing need to be conducted to achieve a more comprehensive assessment of the drug resistance profiles in both CSF and plasma.

In conclusion, we revealed a high concordance of HIV-1 DRMs between CSF and plasma, with no mutations identified in CSF that were absent in plasma. NNRTI-related resistance mutations were predominantly detected, notably the V179D/E mutations, primarily in ART-naive patients, most likely attributable to polymorphisms. These results indicate that when a patient’s ART regimen is adjusted according to the drug resistance profile derived from plasma, the antiretroviral drug would remain effective in the CSF.

## Data Availability

The datasets presented in this study can be found in online repositories. The names of the repository/repositories and accession number(s) can be found at: https://www.ncbi.nlm.nih.gov/genbank/, PV240106-PV240179; PV242912-242985.
